# Skeletal age in idiopathic short stature: An analytical study by the TW3 method, Greulich and Pyle method

**DOI:** 10.4103/0019-5413.65144

**Published:** 2010

**Authors:** Hak Jun Kim, Hae-Ryong Song, Ashok Shyam, Song Sang Heon, Ranjith Unnikrishnan, Sang-Youn Song

**Affiliations:** Department of Orthopaedic Surgery, Rare Disease Institute, Korea University Guro Hospital, Seoul, Korea; 1 Sancheti Institute of Orthopaedic and Rehabilitation, Shivaji Nagar, Pune; Indian Orthopaedic Research Group, Thane, Maharashtra, India; 2 Department of Chemistry, Kung Hee University, Seoul, Korea

**Keywords:** Greulich and Pyle atlas, idiopathic short stature, skeletal age, TW3 method

## Abstract

**Background:**

The skeletal age in short stature and in various other growth abnormalities is well documented. We lack the study pertaining to the analysis of the skeletal age in idiopathic short stature or analyzing the difference in skeletal age delay or advancement between the familial short stature (FSS) and non-familial short stature (non-FSS) groups, hence this study. Present retrospective study is designed to study the variation in patterns of skeletal age in ISS.

**Materials and Methods:**

One hundred and eighty six patients, 95 males and 91 females of idiopathic short stature were examined to assess the skeletal age deviation in relation to chronological age. The radiographs of the left hand and wrist were done. The skeletal age was assessed using Tanner and Whitehouse (TW3) method and Greulich and Pyle (GP) atlas. The patients were divided into two groups based on the parental heights. Group A (Familial Short Stature; FSS) with 100 patients (55 males, 45 females) included patients whose at least one parent was short and Group B (non-Familial Short Stature; non-FSS) with 86 patients (40 males, 46 females), included patients whose parental height was normal. The carpal scores, RUS (Radius, Ulna and Short bone) scores and GP age were determined and the respective delay or advances were calculated.

**Results:**

The skeletal age in Group A was delayed relative to chronological age by a mean of 1.9 years in males and 2.3 years in females (*P*<0.05) by RUS method, mean of 2.7 years in males and 2.6 years in females by Carpal score (*P*<0.05), 2.2 years in males and 2.7 years in females by GP atlas age (*P*<0.05). The skeletal age in Group B was advanced by a mean of 0.9 years in males and 1.4 years in females (*P*<0.05) by RUS method, mean of 0.4 years in males and 0.35 years in females by Carpal score (*P*<0.05), mean of 1.1 years in males and 0.2 years in females by GP atlas method (*P*<0.05). The Pearson’s coefficient of correlation (*P*<.001) demonstrated good agreement association between all three scores.

**Conclusions:**

There is definite age delay in both males and females in the FSS group while the bone maturation is accelerated in the non-FSS group. Both RUS and GP show good correlation amongst both the genders in both the groups and there is good inter observer correlation for both the methods. We can hypothesize that while treatment protocols to accelerate bone age will be beneficial in the FSS group, these should be avoided in the non-FSS group. Our study also indicates that there definitely exists a difference in normal growth curves in both these groups and a detailed study is required to plot their respective normal growth lines so as to make proper adjustments in the assessment of the remaining growth and limb lengthening protocols.

## INTRODUCTION

Short stature is a common clinical condition faced by the pediatric orthopedic surgeon. The evaluation and treatment of these children is strategic especially with regards to the prediction of the adult height and planning of any intervention if deemed necessary. Accurate skeletal age determination and assessment of the variations relative to chronological age is vital to formulate the treatment plan.

Among the many methods proposed for assessing skeletal maturity, those of Greulich and Pyle (GP) and of Tanner and Whitehouse (TW3) are commonly used in clinical practice. The secular trend seen in skeletal maturity has forced the (TW3) system out of favor^1^. Also, significant differences between the RUS and carpel bone age are now well accepted and two separate bone age maturity scores, one each for RUS and carpel bone age, are used in TW3 system. The validity of TW3 method in Asian and Far Eastern population has been supported by Tanner *et al*.^1^ As compared to the GP atlas method the TW3 method is more flexible and has less standard error, since it derives from a more solid mathematical base, but has disadvantages of being difficult to perform and being time-consuming. The pros and cons of both methods have been well discussed in the literature.^2^-^4^

There are many references in literature on the study of skeletal age in short stature and in various other growth abnormalities^5^-^11^ but we could not find any study pertaining to the analysis of the skeletal age in idiopathic short stature or analyzing the difference in skeletal age delay or advancement between the familial short stature (FSS) and non-familial short stature (non-FSS) groups. Our simplified classification is based on the predictive value of parental height as a function of final height attained by these children. The role of parental height in determination of the final adult height has been studied extensively.^8^,^12^,^13^ We designed this retrospective study to improve our understanding about the patterns of skeletal age in patients with ISS and variations of these patterns amongst the two genders and two subgroups. These patterns will possibly help us define parameters to decide for the right kind of intervention and will also shed some light on the natural history of ISS.

## MATERIALS AND METHODS

Two hundred and thirty patients referred to our pediatric orthopedic services with measured total heights less than 3rd percentile line from the Korean growth chart^14^ (Size Korea Report, 2004) were assessed. A detailed clinical examination with investigations including radiographs and hormonal assays for growth hormone was performed to diagnose syndromes, metabolic disorders affecting the bone growth and growth hormone abnormalities. If patients do not have any such abnormalities, they are diagnosed to have Idiopathic Short Stature and then included in our study. Forty four patients were excluded with different primary diagnosis or incomplete records and poor radiographs. Thus a total of 186 patients, 95 males and 91 females who presented between 2003 and 2005 were included in this study. The study group included patients of Korean origin only. The data regarding the height of both the parents were obtained. The parent was considered short if the height was below the third percentile as per Korean standard height.^15^ The patients were divided into two groups: Group A, the FSS group, comprised of patients whose parents (either one or both) were considered short. This group had 100 patients, 55 males (age 4.8 to 18 years) and 45 females (age 4.1 to 17.8 years). Group B consisted of non-FSS group whose parents were of normal height. This group had 86 patients which included 40 males (age 9.2 to 16 years) and 46 females (age 10.3 to 17 years).

All the patients had anteroposterior radiograms of both hands including wrist joints performed. The left hand radiogram was used to determine the skeletal age. Thus a total of 186 hand radiograms in 186 patients were available for assessment of skeletal maturation. The skeletal age was assessed using the TW3 and GP atlas method. The scoring was done by two observers with training in TW3 and GP atlas scoring systems and the mean value was taken. In the TW3 system, a maturity score is assigned independently to each epiphysis of the radius, ulna, 1^st^, 3^rd^ and 5^th^ metacarpals and phalanges and to each carpal bone. The scores are given on the basis of recognizable stages of development through which each bone passes between its first appearance and mature state. Independent maturity scores were obtained for the radius, ulna, metacarpals and phalanges (RUS) and for the carpals. For each carpal and RUS score, the corresponding carpal and RUS bone age equivalents were obtained^1^ The GP age was assessed by comparison of the patient’s radiogram and the corresponding standard in the Greulich-Pyle atlas.^16^

Statistical analysis was done using SPSS software. The student’s t test was employed to assess the significance of the skeletal age variations between both groups with respect to RUS age delay, carpal age delay and GP age delay [Table 1]. The student’s t test was also used to calculate P value separately for males and females [Table 2]. The values of correlation between the RUS age delay, carpal age delay and GP age delay was also determined within both the groups independently [Tables 3 and 4]. Value of *P*<0.05 was considered as significant. ANCOVA test was done for adjustment in variation of chronological age between the two groups and results are presented separately for males and females [Table 5].

**Table 1:** Comparison of RUS delay, Carpal delay and GP delay between Group A and Group B

Variable	Group A (*n*=100)		Group B (*n*=86)		*P* value
	Mean	SD	Mean	SD	
RUS delay	2.10	±1.50	−1.20	±1.10	<.001
Carpal delay	2.70	±1.90	−0.07	±1.70	<.001
GP delay	2.40	±1.80	0.20	±1.80	<.001

All *P* values are for student’s t-test; Group A – Familial short stature group; Group B – Non-Familial short stature group

**Table 2:** Comparison of RUS delay, Carpal delay and GP delay assessed separately for two sexes, between Group A and Group B

Variable	Group A (*n*=55)		Group B (*n*=40)		*P* value
	Mean	SD	Mean	SD	
Male					
RUS delay	1.9	±1.2	−0.9	±0.5	<.001
Carpal delay	2.7	±1.9	−0.4	±1.6	<.001
GP delay	2.2	±1.8	−1.10	±2.3	0.046

**Variable**	**Group A (*n*=45)**		**Group B (*n*=46)**		***P* value**
	**Mean**	**SD**	**Mean**	**SD**	

Female					
RUS delay	2.3	±2.0	−1.4	±1.3	<.001
Carpal delay	2.6	±1.9	−0.3	±1.7	<.001
GP delay	2.7	±1.6	−0.2	±1.3	<.001

All *P* values are for student’s t-test; Group A – Familial short stature group; Group B – Non-Familial short stature group

**Table 3:** Correlation between the RUS age delay and Carpal age delay within Group A and Group B

	Total	Male	Female
Group A			
Pearson’s coefficient	0.673	0.711	0.562
*P* value	<.001	<.001	<.001
Group B			
Pearson’s coefficient	0.644	0.571	0.595
*P* value	<.001	0.052	0.002

All *P* values are for student’s t-test; Group A – Familial short stature group; Group B – Non-Familial short stature group

**Table 4:** Correlation between the RUS age delay and GP age delay within Group A and Group B

	Total	Male	Female
Group A			
Pearson’s coefficient	0.583	0.438	0.822
*P* value	<.001	<.001	<.001
Group B			
Pearson’s coefficient	0.531	0.074	0.779
*P* value	0.009	0.819	<.001

All *P* values are for student’s t-test; Group A – Familial short stature group; Group B – Non-Familial short stature group

**Table 5:** Comparison of A and B groups by ANCOVA test with respect to gender

Groups A and B			
Males		Females	
Delay	*P* value	Delay	*P* value
RUS delay	<0.01	RUS delay	<0.01
Carpal delay	<.001	Carpal delay	<.001
GP delay	0.02	GP delay	<0.01

Figures of *P* value less than 0.01, means the respective delay comparison between A and B group is significant; Group A – Familial short stature group; group B – Non-Familial short stature group

## RESULTS

The mean chronological age of the patients in Group A was 9.1±2.9 years (4.8 to 18.0 years). The mean RUS score was 315±192 (71 to 1000) which translated into a mean RUS bone age of 8.3±3.2 years (2.6 to 16.5 years). The mean carpal bone score was 533±245 (183 to 1000) which corresponded to a mean carpal bone age of 7.6±3.0 years (2.3 to 15.0 years). The mean RUS bone age delay was 2.1±1.5 years (−1.7 to 3.9 years) (negative value indicates that bone age is greater than chronological age) whereas the mean carpal bone age delay was 2.7±1.9 years (−0.5 to 5.3 years). The GP age delay was 2.4±1.8 years.

In Group B the mean chronological age was 12.0±2.2 years (9.7 to 16.1 years). The mean RUS score was 747±234 (338 to 1000) which translated into a mean RUS bone age of 13.2±2.1 years (9.7 to 16.5 years). The mean carpal bone score was 937±97 (666 to 1000) which corresponded to a mean carpal bone age of 12.0±1.6 years (9.4 to 15.0 years). The mean RUS bone age delay was −1.2±1.1 years (−0.03 to −4.4 years) (negative value indicates that bone age is greater than chronological age i.e. bone age is advanced as compared to chronological age) whereas the mean carpal bone age delay was −0.07±1.7 years. The GP age delay was 0.2±1.8 years [Table 1].

In Group A the pattern was of consistent skeletal age delay with the delay varying between 0.1years to 5.2 years. The delay as assessed by all the three methods, carpal age, RUS age and GP age was very significant (*P*<0.001). This delay in bone age was most pronounced in females (*P*<0.001) as compared to males (*P* < 0.001 to 0.046) [Table 2]. The Pearson’s coefficient of correlation between the RUS delay and carpal age delay was 0.711 in males (*P* < 0.001) and 0.562 in females (*P* < 0.001) [Table 3].

In Group B, the female patients showed a uniform acceleration of maturation as measured by all three methods, with average advancement of 1.4 years, 0.3 years and 0.2 years by RUS, carpal and GP method, respectively. The male patients too showed acceleration of maturation of average 0.9 years as measured by the RUS age which was statistically significant (*P* < 0.001) when compared to Group A [Table 2]. This acceleration was averaging 0.4 years as measured by the carpal age (*P* < 0.002) and 1.1 years by the GP method (*P* < 0.046). The Pearson’s coefficient was not significant in males of group B when the RUS delay was compared to the carpal delay (*P*=0.052) [Table 3] and GP delay (*P*=0.819) [Table 4]. However, the ANCOVA test confirmed the overall difference between the RUS delay, carpal delay and GP delay between the two groups to be statistically significant in their respective gender subgroups [Table 5].

Inter-observer studies showed good agreement suggesting that there was reliable correlation between observers and a high level of reproducibility for individual observers. The overall inter-observer concurrence between the two observers for both TW3 and GP method was good with a correlation co-efficient of between 0.85 to 0.95 with a confidence interval between 0.63 to 0.98 and an overall *P* value less than 0.029.

## DISCUSSION

Idiopathic short stature comprises of patients in whom known causes of dwarfism are ruled out. Also the possible causes of pathologic shortness of height like skeletal dysplasias and metabolic causes are excluded. The skeletal development in these patients is believed to be deranged with skeletal age delay of 1-2 years.^7^ However, the exact pattern of this delay of maturation of the skeleton has not been dwelled upon in literature. In our study we attempt to explore the discrepancy between biological maturation and chronological age in patients with idiopathic short stature.

To the best of our knowledge, this is the first study that analyzes the skeletal age delay pattern in idiopathic short stature. We analyzed the pattern of delay by the latest TW3 method and the long established GP atlas method. The findings are consistent with the present thought that there is a skeletal age delay of 1 -2 years in idiopathic short stature as far as the familial short stature group is concerned, but in the non-familial short stature the pattern is reversed with the patients showing an acceleration of skeletal maturation of mean 0.8 years in males and 0.6 years in females (where mean is the mean of RUS delay, carpal delay and GP delay of the two sexes as presented in Table 2). This pattern of skeletal age variance showed good correlation irrespective of the method employed, TW3 or GP atlas methods. This indicates that the results of the GP method which is less sophisticated and the TW3 method are comparable and thus both methods are valid for such studies. Both the methods have shown good inter observer correlation too indicating reproducible results.

Majority of the patients in the study group comprised of familial short stature and this finding is in concordance with the report of Lindsay *et al*.^17^ They were the first to distinguish between the FSS and the non-FSS groups and study the effect of growth hormone on idiopathic short stature. However, their study did not attempt to assess the skeletal age deviation from normal. It is vital according to Bololi *et al*.^9^ to assess the skeletal age in these patients with respect to the validity of intervention like hormone therapy.^18^,^19^ Our study provides this information and finds that FSS group has age delay while the non-FSS group has age acceleration. A very interesting observation noted by Kelnar *et al*. 8 is that when children with ISS are treated with growth hormone, the FSS group achieved their target height, whereas those with non-FSS did not. This can be explained on the basis of our finding showing non-FSS group to have advanced bone age, thus having early physeal closure when treated with growth hormone. Hence, in the non-FSS group, other novel methods like estrogen blocking aromatase inhibitors that delay fusion of physes can be used and growth hormone treatment may be contraindicated in this group.^20^ The outcome of our study indicates a need to make corrections in estimating the final adult height and timing of limb lengthening by straight line graph method of Moseley when dealing with ISS. Our findings suggest that the FSS and non-FSS groups will have different slopes of the normal growth line and this in turn will change the assessment of the remaining height and limb lengthening timing protocols. We postulate that the FSS group which shows skeletal age delays should be preferably treated with medical line of management like growth hormone therapy while the non-FSS group that showed advanced skeletal age and early fusion of the physis should have low threshold for limb lengthening surgeries at an early age. However, a detailed study of the two groups with this respect is required.

Thus, in conclusion, our study suggests that there is definite age delay in both males and females in the FSS group while the bone maturation is accelerated in both males and females in the non-FSS group. In view of these findings, we can hypothesize that use of growth hormone in ISS patients with either one of the parents with short stature is justified however such a therapy in the non-FSS group should be avoided and also that the two groups should be further studied in detail to plot their respective normal growth lines so as to make proper adjustments in the assessment of the remaining growth and limb lengthening protocols. One weakness of this study is lack of long term follow-up of the changes in the skeletal age delay or advancement pattern in idiopathic short stature. However, the results of our study can be guide line to evaluate skeletal age in idiopathic short stature. Further detailed studies are warranted to establish this change in the delay or acceleration of skeletal maturation exhibited by these subdivisions of FSS and non-FSS in our pilot study.
